# Magnesium-Titanium Alloys: A Promising Solution for Biodegradable Biomedical Implants

**DOI:** 10.3390/ma17215157

**Published:** 2024-10-23

**Authors:** Sachin Kumar Sharma, Sandra Gajević, Lokesh Kumar Sharma, Reshab Pradhan, Slavica Miladinović, Aleksandar Ašonja, Blaža Stojanović

**Affiliations:** 1 Surface Science and Tribology Lab, Department of Mechanical Engineering, Shiv Nadar Institute of Eminence, Gautam Buddha Nagar 201314, India; rp943@snu.edu.in; 2 Faculty of Engineering, University of Kragujevac, Sestre Janjić 6, 34000 Kragujevac, Serbia; slavicam@kg.ac.rs (S.M.); blaza@kg.ac.rs (B.S.); 3 Department of Physics, GLA University, Mathura 281406, India; lokesh.sharma@gla.ac.in; 4 Faculty of Economics and Engineering Management in Novi Sad, University Business Academy in Novi Sad, Cvećarska 2, 21000 Novi Sad, Serbia; asonja.aleksandar@fimek.edu.rs

**Keywords:** magnesium, ball-milling, spark plasma sintering, corrosion, biodegradable

## Abstract

Magnesium (Mg) has attracted considerable attention as a biodegradable material for medical implants owing to its excellent biocompatibility, mitigating long-term toxicity and stress shielding. Nevertheless, challenges arise from its rapid degradation and low corrosion resistance under physiological conditions. To overcome these challenges, titanium (biocompatibility and corrosion resistance) has been integrated into Mg. The incorporation of titanium significantly improves mechanical and corrosion resistance properties, thereby enhancing performance in biological settings. Mg–Ti alloys are produced through mechanical alloying and spark plasma sintering (SPS). The SPS technique transforms powder mixtures into bulk materials while preserving structural integrity, resulting in enhanced corrosion resistance, particularly Mg80-Ti20 alloy in simulated body fluids. Moreover, Mg–Ti alloy revealed no more toxicity when assessed on pre-osteoblastic cells. Furthermore, the ability of Mg–Ti-based alloy to create composites with polymers such as PLGA (polylactic-co-glycolic acid) widen their biomedical applications by regulating degradation and ensuring pH stability. These alloys promote temporary orthopaedic implants, offering initial load-bearing capacity during the healing process of fractures without requiring a second surgery for removal. To address scalability constraints, further research is necessary to investigate additional consolidation methods beyond SPS. It is essential to evaluate the relationship between corrosion and mechanical loading to confirm their adequacy in physiological environments. This review article highlights the importance of mechanical characterization and corrosion evaluation of Mg–Ti alloys, reinforcing their applicability in fracture fixation and various biomedical implants.

## 1. Introduction

Biomaterial characteristics are a prerequisite for effective *osseointegration* in biomedical applications with superior biocompatibility and mechanical endurance which is regarded as necessary conditions for permanent and temporary implantation [1]. Multiple implantations involve plates and screws for fixation of internal fractures in which corrosion resistance is the key feature of implantation. The implant materials should have adequate mechanical properties, biological stability, and resemble the bone characteristics in terms of stiffness, flexibility, lightness, and strength, which allows the interaction of hard and soft tissues, blood, extracellular and intracellular fluids in the human body [2,3]. Therefore, implant materials which are highly compatible with the body hold good accountability for biocompatibility with an osseointegration-friendly nature. Metals, ceramics, polymers, and composites are the four major forms of biomaterials used for implantation [4]. Stainless steels, titanium, and cobalt-chromium-based alloys are commonly used metallic biomaterials. Metallic materials other than ceramics or polymers are crucial in providing a good healing rate to tissue, replacing diseased or damaged bone tissue, suitable for load-bearing applications accommodating high mechanical strength, and fracture toughness [5]. Hence, metallic implant materials are employed in a wide range of applications in biomedical applications. The implant material must possess sufficient biocompatibility, bio-activeness, and biodegradability. Biodegradable implants provide the remodelling of bone, tissue healing rate, tabulating the injured bone, and restricting the chances of a second surgery [6]. Biodegradable implant materials rely on polymers and metals that are mechanically strong and have good corrosion resistance [7]. Initially, Mg-based implant material for bone fixation showed excellent resorbability and biocompatibility.

**Figure 1:**
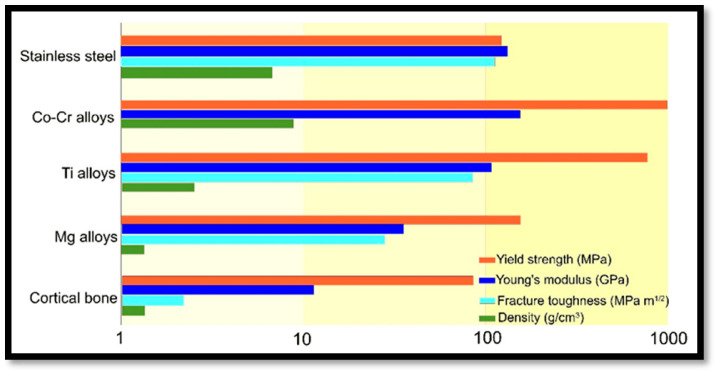
The elastic moduli, density, yield strength, and fracture toughness of several metallic biomaterials are compared to those of cortical bone [8].

Magnesium is considered bioactive, biodegradable, biocompatible, and resembles the elastic modulus of natural bone [7]. The low elastic modulus of Mg-based materials helps to reduce the stress shielding effect, which is a major problem with stainless steel, cobalt-chromium alloy, and titanium-based implant material (Figure 1) [8]. Due to the high value of the elastic modulus, most of the stress is acting on the bone, hindering the new bone stimulation and remodelling. But corrosion of Mg and its alloys are the major areas of concern when dealing with biomedical implantation. Enhancing the corrosion resistance of Mg-based alloys in SBF (simulated body fluid) without affecting its biocompatibility is a serious concern. Titanium is regarded as a suitable reinforcing agent that improves corrosion resistance of Mg based material, maintaining biocompatibility [9,10,11]. The first-generation biomaterials focused on replacing the physical characteristics of tissues with no toxicity to host. The second generation interacts with biological surroundings to promote tissue attachment along with disintegrating the newly generated tissues. The third generation examines the molecular stimulation of specific cell responses pertaining to bone tissue [12].

Biomaterials promote the interaction of cells enabling the regeneration of new bones. Mechanical stresses promote cell function and impact the adhesion of integrins on cell surfaces with respect to the substrate [13]. Mechano-transduction is the process by which cells turn stresses into biological signals, initiated by structural stresses or fluid shear stresses generated in the materials. Pore size, distribution, shape, hydrophobicity/hydrophilicity, time-dependent deformation, chemical functionality, pH level change, temperature and stress, micro-and nanoscale surface topography, and mechanisms of biodegradation are the factors affecting implantation [14]. Artificial implant biomaterials are implemented to relieve pain and restore dysfunctional tissue performance. Stainless steel involves pins, wires, screws, plates, intramedullary nails or rods, among other items, employed in internal fixation devices due to its high strength [15]. But stainless steel releases harmful alloying elements, such as nickel and chromium, which decreases cell viability. Metal-based implants employed in biomedical applications reduce the severe effects of nickel as well as the stress shielding effect [16]. Natural bones have collagen protein, non-collagenous proteins, and hydroxyapatite, carrying large biomechanical loads [17]. However, the unique ability for regeneration from trauma, along with disease and fractures are easily healed without a second surgery [18]. During the tissue healing period, the implant material should gradually disintegrate as the damaged tissue heals, transferring the suitable amount of load to the body until the injured bone has totally recovered [19]. The corrosion rate should be low enough not to allow distortion or fracture under the application of load. The large variation in the elastic modulus of bone and implant material causes a serious threat to high-stress shielding protection [20]. The stress shielding mainly occurs due to bone absorption, leading to implant loosening, or refracture of bone tissue. Therefore, the elastic modulus should be similar to the implant material, lowering stress shielding protection and entailing a good tissue healing rate.

Biodegradable implantation is preferred for rapid tissue healing rate to bone tissue along with regeneration of tissue bone [21]. Magnesium (biodegradable material) is a promising material for biomedical applications offering high cytocompatibility, superior mechanical properties, and an elastic modulus similar to natural bone [22]. Mg can be employed in bone and cardiovascular applications for restoring fracture. Magnesium-based implants provide initial stability, load-bearing support to the implant before degraded in vivo, thereby terminating the need for additional surgery after implantation [20,21,22]. However, the corrosive nature of magnesium-based alloys enables direct contact with bone offering a good link between magnesium and bone [23]. Therefore, implant integration into the bone is critical, dependent on the bone–tissue–implant interface [24]. The degraded magnesium restored in the callus covers the bone fracture site and is absorbed into the bloodstream and expelled through urine which causes renal failure and poor kidney function [25]. Further, magnesium tends to generate gaps at the bone-implant interface due to its rapid deterioration. Hence, avoiding magnesium breakdown in vivo is a challenging task but a suitable alloying element can control rapid deterioration of magnesium in bone.

Due to its light weight and low density, magnesium has excellent mechanical strength and fracture toughness, but significantly poor corrosion resistance [26]. Large volumes of hydrogen are absorbed by magnesium and its alloys, used as fuel cell hydrogen storage materials and as magnesium hydride electrodes for nickel-hydride batteries [27]. With low corrosion resistance and high susceptibility to oxidation, the usage of magnesium and its alloys is restricted. Metal implants, ceramics, and polymers have a high elastic modulus and provide shear protection in bone. Magnesium has a better fracture toughness when used in bone formation, orthopaedic, and bone regenerative applications [28]. Since magnesium corrodes easily, the pH level around the implant rises and hydrogen bubbles are produced, separating the surrounding tissue of the body and impeding the healing process [29]. Mg alloy as biomaterial for implantation was to be a bioabsorbable coronary stent. Magnesium is naturally non-toxic, thus when used in biomedical applications, it does not create any inflammation [30]. Mg^2+^ is a readily available intercellular cation that acts as an abundant cation in the body, contains half of its magnesium in soft tissue and the other half in bones [31]. Magnesium is crucial for bone metabolism and osteoclasts and provides bones the strength to reconstruct [32]. Numerous chronic illnesses occurred due to magnesium shortage in the body. Magnesium shortage results in high blood pressure in the cardiovascular system, atherosclerosis, coronary artery vasoconstriction, and cardiac arrhythmias. Magnesium increases the cardiac index by reducing vascular resistance [33,34]. Therefore, Mg is considered a vital and crucial material for biomedical application, but corrosion and wear aspects are again questionable. Therefore, Titanium added to Mg-based materials paved the way to improve the implantation healing process and bone tissue recovery, as titanium improves the wear and corrosion resistance of Mg-based materials. The current review article deals with the in-depth aspects related to Mg–Ti alloy relating to mechanical and corrosion aspects, governing the scope of Mg–Ti alloy in biomedical implantation.

## 2. Corrosion Behaviour of Magnesium Materials in a Biological Environment

Because of their outstanding strength-to-weight ratios and low densities, magnesium alloys are ideal materials; nevertheless, their weak corrosion resistance limits their applicability [35]. Magnesium has the least standardized potential (Ca, Na, Li, and K) among all engineering metal (−2.38 V versus Standard hydrogen electrode (SHE)) and corrode in physiological situations [36]. Magnesium is commonly employed as a sacrificial anode to safeguard cathodic metals because of its extremely low potential [37]. In a dilute chloride solution, magnesium has a corrosion potential of −1.7 V vs. SHE [38]. Higher pH values significantly minimize corrosion; hence the pH value is crucial. Due to the sensitivity of magnesium corrosion, an increase in pH levels brought on by magnesium corrosion in vitro testing significantly affects the environment, which causes data to be fudged [39,40,41]. The magnesium corrosion rate falls monotonically with increasing pH, attaining complete passivation around a pH of 12. Protective covering of Mg(OH)_2_ formed on outside surface persists at a pH level of 11.5 in solution that is alkaline, low levels of corrosion are attained [42]. The same issue happens when a salty test sample is improperly buffered. To avoid any pH variations, a corrosive solution should be buffered by 2-(4-(2-hydroxyethyl)-1-piperazinyl) ethane sulfonic acid [43]. By contrast, alkalization happens in real life, hence eliminating pH shifts could lead to artificial surroundings that do not truly represent physiological situations. Comparing quantitative results, however, is much simpler. Fixing the pH also stops some unavoidable effects, including salt deposition, which happens as the pH level rises [44].

**Figure 2:**
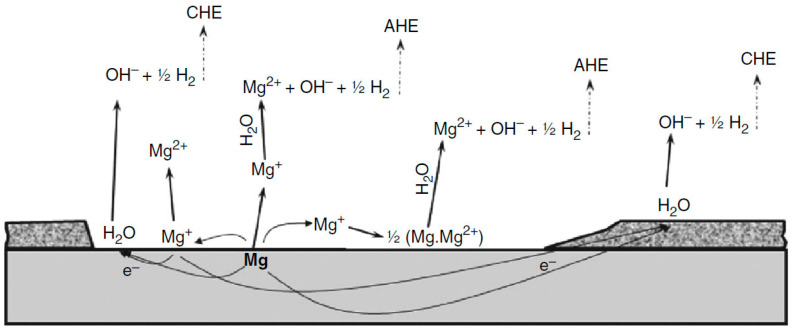
The anodic and cathodic reactions that are involved in the self-corrosion of magnesium are symbolically shown [50].

Magnesium has a weak resistance to corrosion in aqueous media because of its strong tendency for dissolution at pH levels below 11, its permeability as well as the low stability in the magnesium hydroxide covering that form on its exterior, and its reactivity to alloying metallic substances and contaminants that encourage galvanic corrosion [45]. The main factor influencing how magnesium alloys respond to corrosion is the alloying component [46]. Due to a micro-galvanic interaction between the secondary phase and matrix, magnesium alloys with two phases corrode more quickly [47]. The phase distribution, size, and grain boundary are examples of microstructural features that affect how magnesium alloys behave tribologically [48,49]. In addition to mechanical qualities, corrosion rate is impacted by grain improvement, which alters both the density and dispersal of grain boundaries. Magnesium corrodes more quickly in water-based solutions but more slowly in sulphate as well as chloride ions (Figure 2) [50]. Both magnesium hydroxide as well as hydrogen gas are produced when water molecules in aqueous solutions are reduced, as demonstrated by the equation below [51,52]. Hydrogen bubbles arise on Mg in aqueous solutions when corrosion occurs because magnesium’s potential is lower than the water stability range at neutral or acidic pH levels [53,54]. The by-product of the ions of hydroxide raises the pH. The existence of hydroxide ions in the environment promotes the synthesis of Mg(OH)_2_ persistent at high pH levels controls the kinetics of corrosion [55,56].

At cathode: 2H_2_O + 2e^−^ → 2OH^−^ + H_2_ (1)

At anode: Mg → Mg^2+^ + 2e^−^ (2)

Overall reaction: Mg + 2H_2_O → H_2_ + Mg(OH)_2_ (3)

The surface coating is created by the following chemical precipitation reaction:

Mg^2+^ + 2OH^−^ → Mg(OH)_2_ (4)

**Figure 3:**
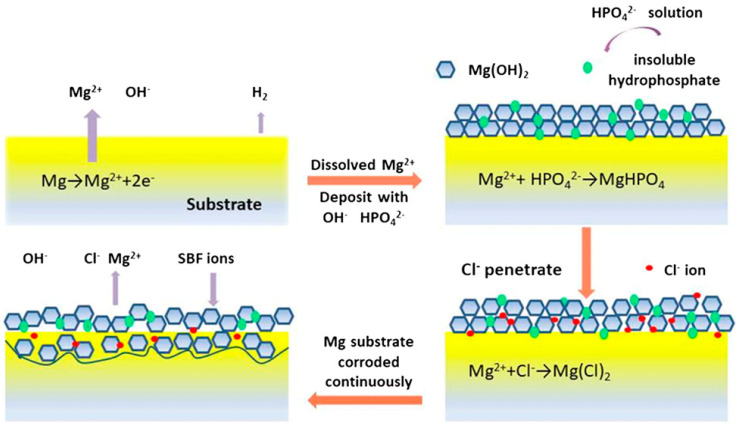
A graphical representation of the mechanisms that cause corrosion in magnesium-based alloys when they are exposed to a simulated body fluid (SBF) solution [59].

The build-up of soluble corrosion-related substances on the surface, such as the magnesium hydroxide in magnesium alloys, impedes the charge transfer process [57]. While magnesium corrodes as a result of saturation and localized alkalization, corrosion by-products form precipitates from the test solution [58]. Magnesium hydroxide is not very soluble in water. On the other hand, chlorine ions readily shatter this mineral’s hexagonal structure, turning it into MgCl_2_ (Figure 3) [59]. Since magnesium chloride dissolves readily, the surface layer degradation is accelerated by the addition of Cl ions [60]. Equations (5) and (6) show how magnesium reacts with saline solutions to form hydroxides, chlorides, and oxides. In solutions with a pH < 10, there should not be any surface coating on Mg (Pourbaix diagram) as the film would be unstable [61].

Mg(OH)_2_ + 2Cl^−^ → MgCl_2_ + 2OH^−^ (5)

Mg → Mg^+^ + e^−^ (6)

Mg^+^ is a transient ion that is imperceptible in ring-disk electrode experiments [62]. The chemical reaction produces hydrogen from the monovalent magnesium ion as depicted in Equation (7),

Mg^+^ + 2H ^+^ → 2Mg^2+^ + H_2_ (7)

The production of magnesium hydride was also suggested as a mechanism as depicted in Equation (8). Magnesium hydride is unstable when exposed to water. It then interacts to form hydrogen gas, according to thermodynamic data as depicted in Equation (9),

Mg + H_2_ → MgH_2_ (8)

MgH_2_ + 2H_2_O → Mg^2+^ + 2OH^−^ + 2H_2_ (9)

Mg(OH)_2_ and MgO formed an outer layer of corroded Mg using ex situ methods [63]. The existence of MgH_2_ was demonstrated residue that was scraped from a magnesium electrode’s surface [64]. Magnesium hydride might be the source of brittle fracture since it may be brought on by the embrittlement of hydrogen across the boundaries of grains and dislocations [65]. For materials based on magnesium, reports of granular corrosion, pitting, and filiform corrosion have all been made [66]. Corrosion may also be caused by fatigue. De-alloying and particle undermining are two corrosion mechanisms. Magnesium hydroxides and hydrogen create a thin layer when magnesium separates on the surface of the water, but hydrogen stress cracking causes the magnesium-containing particle to physically separate [67,68]. When magnesium ions in the air come into touch with water, the layer of oxide that develops on the metal transforms into Mg(OH)_2_ [69]. This causes the magnesium hydroxide layer to thicken, lowering the potential and increasing the film’s protective qualities. Consequently, there is an increase in the rate of hydrogen evolution and magnesium dissolution. The anodic current flowing through an electrode at rest potential decreases with time due to the build-up of ions of magnesium in the layer near the electrode’s surface [70].

Local anodes and cathodes in a free corrosion environment interact to cause magnesium corrosion (no external potential or current supplied) [71]. Below 0.5 mA/cm^2^, Mg has a protective covering to prevent it from dissolving. The impurity-containing cathodic phases are thus exposed to chemical dissolution [72]. The protective coating is damaged over the threshold current density, exposing the electrolyte to impurities as well as magnesium [73]. Magnesium has a comparable electrochemical activity across a pH range from 3 to 11 [74]. The surface coating only covers minor sections of magnesium when moderate anodic currents are applied. The film-free area in acidic solutions is large compared to alkaline solutions depicting that the film dissolves more quickly at lower pH values [75]. Magnesium ions are more likely to deposit and aggregate on the exposed surface layer in alkaline solutions [76]. Consequently, the passive potential is diminished by acidic solutions. When a large provided current or voltage is supplied and considerable amounts of Mg and OH ions are produced at top the electrolyte layer with covered area drops to zero irrespective of bulk pH [76]. The Mg potential in various pH environments remains the same when an outside high anodized current density is applied [77]. When the applied current or potential is negative, the whole surface of the magnesium is coated in a protective coating; the potential of the magnesium is determined by its surface resistance [78]. Experimental studies show that the anodic Mg disintegrating current can increase the corrosion rate more quickly than predicted based on the polarization curve [79]. Magnesium-based materials have an unusual corrosion behaviour when compared to other commonly used metals. The negative difference effect (NDE) is a term the researcher Song used to characterize the various corrosion mechanisms, depicting that magnesium has (NDE) [80]. Research studies based on NDE suggested that the rate of hydrogen evolution in magnesium improves as the anodic polarization increases [81]. The reverse would be expected for the majority of components.

When metal implants come into contact with biological systems, they corrode [82]. Corrosion occurs when metal is oxidized by redox reductions in tissue fluids [83]. Ti reacts fast with H_2_O and forms a thin oxide coating on the surface that shields the metal from further corrosion [84,85]. On titanium surfaces, human osteoclast precursors grew and differentiated into mature osteoclasts [86]. The titanium surface was immediately eroded by mature cells, which absorbed metal ions generated throughout the corrosion process [87]. Once metal ions come into contact with bone, they may be released and cause osteolytic lesions. Sources of magnesium exposure include proteins, blood, dissolved oxygen, and ions like K^+^, Na^+^, Mg^2+^ and Ca^2+^ in the body, organic molecules with low and high molecular weights [88]. According to experimental evidence by Di Virgilio et al., cell metabolism affects corrosion-rate as seen by the difference between the corrosion rates of magnesium particles with and without cells [89]. Hydrogen bubbles appear in the areas of corrosion. Hydrogen gas can be problematic since it is harmful to bodily tissue [90,91,92]. The increase in the local pH of hydroxyl groups remain critical. High pH levels impede down tissue growth and cell multiplication [93,94]. Shear movement and buffers in physiological fluids can both stop the corroding surface’s pH level from rising [95]. However, as the flow rate increases, so does the rate at which biodegradable materials deteriorate. Stress-corrosion of moving liquids can cause cracking, pit growth, and fatigue corrosion, among other mechanical effects. The strength of the implant must also be assessed as it corrodes [96]. Kirkland, Birbilis, and Staiger revealed test standard improving corrosion tests for Mg based biodegradable implantation [97]. Walker et al. [98] looked at how in vitro testing on magnesium alloy corrosion characteristics may be used to predict the rate of corrosion in vivo more accurately. Temperature, the amount of CO_2_, shaking the solution, and simulating fluid exchange by replacing roughly half of it every day were all used to recreate the physiological environment [98]. The process that produced the in vitro corrosion rate for the four magnesium alloy samples under investigation was the nearest to the actual in vivo corrosive rates, even though it was still quicker than that in vivo. Eliminating the corrosion layer that accumulates on the outermost layer before weighing remains a challenge for evaluating mass loss [99]. On the magnesium surface, a layer of hydrated corrosion causes a net weight gain. This rust coating compromises the mechanical integrity of the underlying substrate [100,101,102,103,104,105,106]. Kirkland et al. [97] found that surface-to-medium volume exceeding 50 mL/cm^2^ did not have an effect on mass loss; it was advised to maintain the greatest test solution volume feasible. A rise in pH level results in the precipitation of magnesium and the formation of Mg(OH)_2_ layers on the topmost layer that reduces the susceptibility of corrosion.

When the milling duration is increased at room temperature, the corrosion potential grows nobler. Grosjean et al. revealed that ball milling resists the corrosion of Mg in alkaline aqueous solutions by forming a barrier of protection for magnesium hydroxide hindering formation of grain boundaries and defects over the surface [107]. Processed magnesium has a larger initial potential; a noticeably high potential rises as compared to unmilled magnesium. The development of the passivating Mg(OH)_2_ over the electrode surface is responsible for the change in OCP. The higher OCP variance for ground magnesium might mean that the surface electrolyte reactions are producing more magnesium hydroxide. Ball milling appears to have a good effect on magnesium corrosion resistance, based on the higher potential [105]. With longer milling durations, the anodic current starts at lower potential, increases more quickly for ground Mg, and then is drastically reduced [106] leading to the development of oxygen and the potential simultaneous disintegration of the passivating layer. Longer milling times result in higher OCP and polarization resistance and lower corrosion current density [108]. Conversely, the apparent breakdown potential reduces with milling, suggesting that the passivating film is more readily ruptured or that the thinness of the passive film allows for greater electrical transmission through it, hence promoting electron tunnelling.

In circumstances of more active corrosion (0.3 M borate buffer solution, pH = 8.4), both milled and unmilled Mg revealed active–passive transitions having a pseudo-passive zone; however, the dissolving current of milled magnesium is significantly low [109]. Conversely, milling in borate solution has minimal impact on the polarization resistance and little influence on the corrosion current. Mg corrosion is regulated by cathodic decrease of water, which is insensitive to ball milling. The outermost layer of both milled and unmilled magnesium is MgO [110]. The metallic state is also represented as a peak, indicating that a thin coating of surface oxide is required. The intensity difference between the elemental and oxidized states suggests that there are fewer MgO layers on top of milled magnesium than there were before milling [111]. Peaks of oxide and hydroxide form in both unmilled and milled magnesium upon contact with water [112]. Even though magnesium transforms into Mg(OH)_2_ under water, magnesium oxide is thermodynamically more responsive to magnesium than water; this may account for some of the metal’s higher corrosion resistance [113]. On the other hand, the nanostructure of milled magnesium plays a significant role in its resistance to corrosion.

## 3. Advancements and Modifications in the Corrosion Resistance of Mg

Mg–Ti alloy phase diagram governed reduce solubility of titanium in magnesium prior to positive mixing temperature, high difference in melting temperature, and no intermetallic compounds formed [114]. Non-equilibrium processes are used to produce Mg–Ti alloys such as vapor quenching [33], physical vapour deposition (PVD) [34], high-energy ball milling [114], and magnetron sputtering [115]. A non-equilibrium process increases solubility of Ti in Mg. The most common fabrication methods for metastable supersaturated Mg–Ti alloys are mechanical alloying and physical vapour deposition [116]. Mg–Ti alloy revealed high dislocation density, high residual stress, and small grain size as indicated by XRD pattern [117]. The precipitation occurs during hot pressing or annealing of metastable Mg–Ti alloys at temperatures exceeding 500 °C [118]. By annealing below 200 °C (1 h), the diffraction pattern remains unchanged. When annealing at 300 °C and 350 °C, high intensity of Ti alloy peaks. When the temperature is lower than 500 °C, the grain growth is non-existent [119]. The Mg–Ti alloy was stable at low wt.% of Ti prevents the expansion of the grain and allows the grain boundaries to be refined [120]. The sluggish release of strain below 300 °C was caused by the pinning effect reducing the grain boundary due to interaction of titanium and magnesium around the boundary. Although, the yield strength reduced with improvement in ductility when the titanium volume is increased. The basal texture of the composite along the extrusion direction reduced signifying a reduction in mechanical characteristics [121]. Esen et al. [122] used hot rotary swaging to create biodegradable and bioinert Mg–Ti alloy composites, interlocking Mg in titanium and regenerated bone tissue for better development. The galvanic coupling showed thin oxide layering over Mg surface, separating Mg–Ti contact from an insulating MgO layer by allowing Mg and Ti particle interaction [123,124]. Zheng et al. [125] formed Mg–Ti alloy by vapour quenching creating a supersaturated solid solution. Herringbone patterns and parallel streaks are produced by the columnar grains and sub-grains in the microstructure. Each sub-grain generates dark and bright contrasting zones, misoriented in the [0001] position inside each grain by 5° [126]. Vermeulen et al. [127] formed Mg–Ti alloy by vapour deposition with differing weight fraction of Ti (10 wt.%, 20 wt.%, and 30 wt.%) in Mg composites. Further, Mg–xTi composite formed a hexagonal structure but after hydrogenation Mg70Ti30 and Mg80Ti20 alloys showed FCC, and Mg90Ti10 body centred tetragonal structure.

### 3.1. Ball Milling: Processing Approach

**Figure 4:**
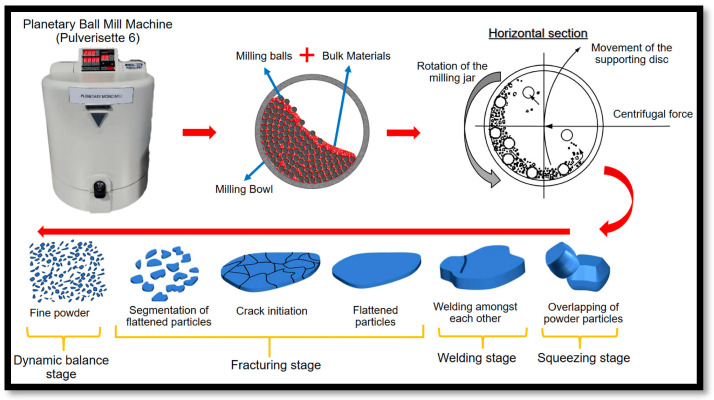
The different stages involved in the ball milling approach, which is widely used for the reduction in the particle size of the powder [140].

Mechanical alloying is used to create alloys and advanced materials at room temperature. Initially, the term milling applied to dissolving large particles into smaller ones, but now creates new phases and materials with enhanced physical and mechanical properties. However, the non-equilibrium approach of high-energy ball milling for nanoscale micro-structured materials has received a lot of attention [127]. Amorphous metals can be produced through mechanical alloying that exceeds the thermodynamic equilibrium concentration [128]. Amorphization occurred with crystal lattice defects and mechanical alloying. Ball milling produced materials, including oxide strengthened alloys (Ti-base alloys, Ni-base superalloys) [129], composites (ceramic-matrix composites, metal-matrix composites) [130], non-equilibrium phases (quasi-crystalline materials, amorphous alloys, nanocrystalline materials) [131], and equilibrium phases (intermetallic compounds, solid solution alloys) [132]. When powder particles are polished to nanoscale dimensions during mechanical alloying, super saturated solid solutions and finely grained microstructures are produced [133]. By continuously cold welding, cracking, and crumpling material involving microstructural and chemical homogeneity, high-energy ball milling leads to alloying at atomic scale [134]. Asano et al. [135] revealed the crystal structure of powders altered by modifying the milling parameter, namely materials used for milling balls as well as pots. In addition, blending, mixing, particle shape, and particle size reduction are accomplished using milling [136]. Both high-energy ball mills (centrifugal, planetary, and vibratory kinds) and low-energy tumble mills with balls or rods can be used for mechanical alloying [137]. To serve as a binder, one constituent possessed moderate ductility, the remaining elements comprised brittle or ductile metals, non-metals, refractory compounds, or intermetallic compounds [138]. For homogenization of materials, the critical factors are particle size distribution, amorphization, level of disorder, final stoichiometry, grinding device (high or low energy) [139]. The materials employed in the fabrication of milling tools, such as tungsten carbide and stainless steel, along with the types of milling media utilized (either rods or balls), the milling environment (comprising inert and reactive gases), the conditions under which milling occurs (wet or dry milling), the mass ratio of powder to milling media, as well as the milling temperature (cryogenic or uncooled) and duration, are all critical factors in the milling process [139,140]. Size of the powder particles has impacted milling time. If they are too coarse then precise finish takes longer. Vibrating and planetary ball mills are employed in mechanical alloying (Figure 4). Fine particles rapidly react with nitrogen, oxygen, and hydrogen in the milling atmosphere because of their high surface-to-volume ratio [140]. This phenomenon was employed for reactive milling only.

In milling, kinetic energy transmits from ball to vial during contact leading to a rise in temperature (100–200 °C) [141]. The input of kinetic energy has a major effect on the degree of amorphization or alloying. The high ball impact rates increase the possibility of tool contamination. The ductile material coated onto the tools limit contamination and prevent subsequent cycles from exposing the milled particles to the bare tool surfaces [142]. Contamination-free processing ensures solid solutions develop without nitrides or oxides reacting chemically, essential for mechanical alloying [142]. Amorphization or alloying was affected by the rate of collisions between wall and balls by powder particles. As a result, formation of amorphous phases rises with the ball to powder mass ratio because kinetic energy is converted to heat, the metastable material overshoots the optimal ball-to-powder ratio and reverts to a regular crystalline state. Finer particles are produced at milling process when the ball and powder ratio is high, which raises contamination caused by the milling tools [143]. This methodology works well with materials that crack when subjected to low temperatures.

Mg–Ti binary alloys are prepared via mechanical alloying in an Ar atmosphere with 10:1 (ball to powder ratio). The enhanced dissolution of Ti particles in magnesium in the nanocrystalline Mg–Ti alloy occurred. The isomeric structure found in titanium and magnesium is responsible for greater solubility [144]. While the reduction in the diffraction intensity, the peaks of the Mg phase also change with mechanical alloying. There were no Ti peaks after 5 h of milling while alloying had taken place [145]. After only one hour of milling, the titanium barely kneads in Mg, while the majority of titanium dissolve in 31 h. Despite having a nominal composition of 10 wt.% and 20 wt.% of Ti, it is projected that, once the milling process entails the stable state based on the lattice properties, only 8 wt.% and 12.5 wt.% titanium will be dissolved in Mg. In the Mg80-Ti20 alloy milling for 12 h, a small, broad titanium peak can be seen at a 40° angle in the XRD spectra. After the milling time is increased to 25 h, no similar peak is formed. A function of milling duration is described as micro-strain obtained in the ball-milling process. The size of the crystallites reduces as the milling duration increases [146]. The size of the crystallites or the micro-strain is unchanged by increasing the milling duration. Kalisvaart et al. [147] used a ball milling approach to induce two FCC phases employing powdered reacting substances, and control agents to make Mg–Ti alloy with composition range varied from 65 vol.% to 85 vol.% of Mg. A hexagon solid solution containing Mg–Ti alloy is produced by the milling process when utilizing a coarse Mg precursor. Ball milling use to partially convert Mg–Ti alloy into more than one FCC phase [148]. Nevertheless, the complete conversion of the binary Mg–Ti composition in steel vials was hindered by the formation of considerable cold-welding. The use of carbide tungsten balls along with a ball-to-powder proportion of 16:1 allowed the binary Mg65-Ti35 alloy to completely convert into the FCC phase [149]. The HCP-to-cubic conversions more strongly affected by the presence of impurities existing in the initial reactants, and unaffected by the presence of stearic acid [150]. Research studies suggested that the binary Mg–Ti alloys resembled three different structures (crystal) when processed via the ball milling approach with Mg concentration (35 vol.% to 80 vol.%) [151,152]. By changing the material and design of the milling tools, FCC, BCC, and HCP shapes were produced [153]. While Ti deforms as a result of twinning, Mg deforms mostly as a result of basal plane slip during ball milling [154]. As Mg content increased in all crystal forms, the lattice parameters also increased because of the larger atomic radius of magnesium as compared to titanium. After the ball milling of Mg with Ti particles, the peaks for titanium disappeared showing Ti is arbitrarily incorporated into the lattice structure without changing the configuration of the crystal [155]. Researchers were able to create FCC alloys with 35 vol.% and 50 vol.% of Mg as well as a blend of FCC and HCP phases with 65 vol.% and 80 vol.% of Mg when they switched to zirconia milling pots and balls [156].

Phases rich in Mg are those with greater lattice values, while phases rich in Ti have smaller lattice constants [157]. The lattice parameter that complies with the BCC structure and HCP of the Mg–Ti alloy follows Vegard’s rule. The stacking faults are brought by dislocated sliding across the basal and prismatic shape planes that constitute the HCP lattice [158]. Although it is hypothesized that a large number of stacking faults were added to the substance in ball milling. According to Asano et al., stacking faults caused the HCP structure of the original powders to be disturbed, resulting in the FCC phase [135]. In Mg and Ti, the twin boundary energy is similar to the stacking fault energy [159]. The persistence of stacking faults connected to the breaking of dislocations is comparable twin boundaries (Mg and Ti) [160]. Compared to zirconia milling tools, stainless steel milling tools are projected to introduce a larger density of stacking defects due to the higher dynamic energy input. The FCC structure changed into the BCC structure as a result of strain-induced nucleation brought on by the development of stacking defects when employing stainless steel milling tools. By adding magnesium to the lattice during ball milling, titanium underwent a phase transition to BCC phase (high temperature) [161]. The crystalline size response to mounting fault density was smaller in BCC than FCC. The mounting fault density is most likely to blame for the shift in crystal structures [162]. Wilkes combined the mechanical alloying process in an inert environment to create a Mg–Ti alloy [163]. This alloy demonstrated the need of eliminating any contamination to obtain a high proportion of Mg with Ti. During the first steps of mechanical alloying, layers of one metal and various ratios of the other element in a solid solution made up a lamellar structure. Mg and Ti were fused together and then sheared and shattered. After processing, the mixture is homogenous and loses its atomic lamellar structure. The governing precipitation of Mg particles considerably enhances the hardness of Mg–Ti solid solutions. The outcome revealed that magnesium drops are stable in Mg–Ti alloys that have been heat-treated and vapor-quenched, which was predicted to allow hot work to solidify the mechanically alloyed powder without changing the material’s characteristics. Using high-energy ball milling, Maweja et al. [150] created a solid combination of Mg and Ti with proportions ranging from Mg44-Ti56 to Mg50-Ti50. Before the experimental material was added, vials milled Ti particles avoid contaminants from the stainless-steel vial. As a result, the surfaces of the ball and vial developed a titanium coating. Traces of the HCP phase rich in Ti were present along with the FCC and BCC structures generated by the Ti–Mg solid form matrices. The twinning in the composition of the Ti–Mg alloy may indicate that strain-induced martensitic transitions from the metastable FCC lattice to the BCC lattice.

Mechanical alloying or highly energetic ball-milling of nanostructured magnesium material is more appropriate for materials intended for hydrogen storage [164,165,166,167]. The natural oxide coating on magnesium is removed during ball milling, exposing fresh active surfaces for the absorption of hydrogen [168,169]. There may be several defects during milling that could serve as hydride nucleation sites. High grain boundary concentrations in nanostructured materials make it easier for hydrogen to enter material [170]. Grosjean et al. [171] analysed the effects of ball-milling upon the composition of chemicals, morphological structure, and electrochemical behaviour of magnesium material using milling periods ranging from 30 min to 40 h. They revealed milling had an impact on the morphology of magnesium material. The process of cold welding, which happens concurrently with the milling process’s frequent particle breakage, creates bigger particles [172]. Particle size generally decreases with longer milling times [173]. Although cold-welding requires a long milling process, the resulting magnesium powder does not include very small particles. Milling has no effect on the position of the diffraction peak, proving lattice properties remain stable, attributing to no contamination of the milling tool as well as no formation of alloy [174].

When little deformation energy is present during the milling and cold pressing of untreated magnesium powder, slip dislocations produce the majority of the deformation, resulting in the {002} crystallographic texture [175,176]. A large amount of energy accumulates in the powder during extended milling (more than three hours), allowing dislocations to travel in multiple directions and leading to randomized deformation [177]. During prolonged milling (over 3 h), a lot of energy builds up, allowing dislocation motion causing randomized deformation [178]. Magnesium that has been milled exhibits widening diffraction peaks, a sign that the crystallinity has drastically decreased [179]. The crystallite size does not fluctuate much as the milling time is extended, entailing that a stable state has been attained [180]. Mg can be converted into nanocrystalline form via ball milling [181]. Magnesium has a lower melting point than other metals which results in larger crystallite sizes after ball milling, preventing plastic deformation [182]. After extended milling, the internal strain increases along with the reduction in crystallite size, which is linked to the distortion in the lattice structure around the grain boundary, increasing the dislocation density [183]. Magnesium has a significant ability for recovery during milling, as evidenced by the fact that the largest strain after 40 h of milling is just 0.056%. The lack of MgO enrichment on the magnesium’s surface indicates homogenous distribution instead of concentrating on surface [184].

### 3.2. Spark Plasma Sintering (SPS): Technique

**Figure 5:**
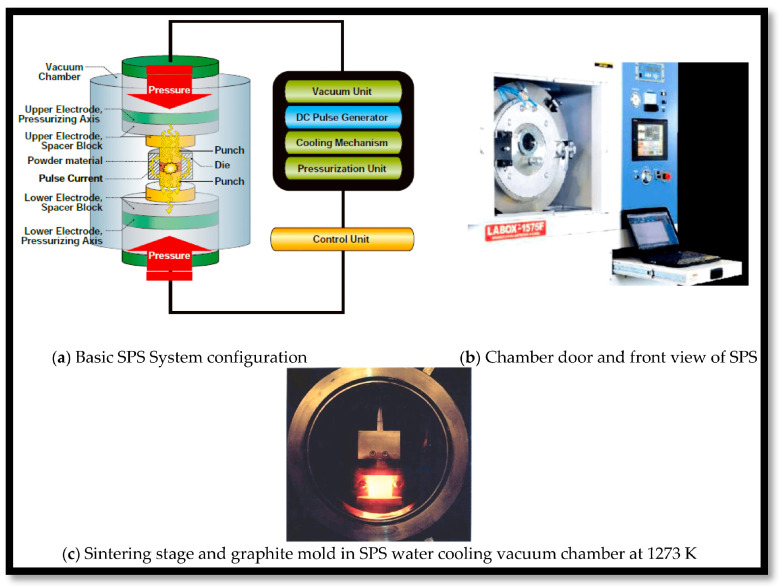
The fundamental configuration of the SPS system includes the following: (**a**) the system configuration; (**b**) the chamber door and a front view of the SPS apparatus; and (**c**) the sintering stage and graphite mould in the SPS water cooling vacuum chamber at 1273 K [189].

The spark plasma sintering method was created to produce bulk materials from powders [185]. Another name that relates to the electric field employed during sintering is electric field-assisted sintering (EFAS). Electric pulse-assisted consolidation (EPAC), plasma-activated sintering (PAS), pulsed electric current sintering (PECS), and field-activated (or assisted) sintering (FAST), are various other names for the same technique [186]. For both nonconductive and conductive materials, SPS can combine a wide choice of complex materials in addition to sintering metals (such as hard and titanium-based alloys), semiconductors, ceramics (such as bio-ceramics), and composites, SPS can also consolidate polymers (such as thermosetting polyimide), interconnect metals, even initiate chemical reactions, and encourage crystal growth [187]. Additionally, it has been used to sinter materials that are difficult to sinter using conventional sintering approach, such as intermetallic compounds, amorphous and nanostructured materials, porous materials, functionally graded materials, highly refractory metals, and metal and ceramic matrix composites. SPS is a highly beneficial method for preserving the nanostructure while generating bulk samples with a certain form [188]. At a single processing step, SPS demonstrates how quickly the powder becomes a full component by generating net and almost net shapes of symmetric and relatively basic components. The primary features of SPS are the sparking plasma produced by a pulse current, together with its quick heating at rates up to 1000 °C/min and brief holding periods (often 0–10 min) (Figure 5) [189]. Research has shown that short processing times combined with low temperatures prevent grain development [188,189,190,191]. SPS may compress nanoparticles of metal with shielding oxides on their surfaces because surface variables play a major role in the creation of inter-particle necks [192]. Oxide-coated powders are insoluble in heat and cannot be sintered using conventional sintering techniques [193].

According to Omori, heat cannot dissolve or remove the oxide layer covering aluminium particles, so traditional sintering methods cannot be used. The oxide layer remains after SPS processing and cannot be entirely eliminated. However, the oxide film can have holes created by the plasma, increasing the heat diffusion capacity of the sintered material in SPS [194]. Particle and grain growth is constrained by the quick cooling rates, the quick process, and the fact that only the surface temperature of particles increases [195]. Precision control of grain formation and microstructure is made possible by quick processing in conjunction with low sintering temperatures, which also preserves particle characteristics and ensures good homogeneity [196]. As with all sintering processes, grain growth happens during SPS. As an outcome, the samples show outstanding levels of consistency and homogeneity in density. The spark plasma has low energy just at the particle contact locations, but when combined with the tension, microscopic holes are made within the oxide layers, which allows small particles to fuse by atomic diffusion [197]. In addition, adsorptive gas and other organic contaminants are removed from particle surfaces by the spark discharge. Because the material with higher purity is exposed to oxide coatings and pollutants, and impurities from particle surfaces evaporate during SPS, greater bonding among particles is produced. SPS is even capable of sintering different materials with lower melt points without the need for a liquid phase [198]. Even at low densities, uniformity is beneficial when creating composites. Spark discharge initiating in the spaces between the particles is facilitated by fine contaminants and gasses [199]. Temperatures as high as 10,000 °C can be locally produced by spark discharges, which evaporate surrounding particles’ surfaces and contaminants [200]. The underlying material melts, taking electrons during the current on phase and producing a vacuum during its off phase, bringing liquidized surface forming necks [201]. Radiant heat can also cause plastic deformation of the particle surfaces, leading to high densities [202]. Researchers claimed that the spark plasma is created in minuscule gaps that electric discharge generates [203]. Local spark impact pressure and the electric discharge combine to generate spark plasma. The spark plasma’s mechanical pressure has an effect on the process, but it is not particularly noticeable.

SPS works better than hot pressing and hot isostatic pressing for sintering [204,205]. SPS has the advantages of regionally shattering the oxide layer, permitting adhesion between particles through the oxide layer’s holes, and enabling full density sintering of even difficult-to-treat materials using traditional sintering techniques [206]. Several factors make SPS a particularly quick densification method, including quick heat transfer, a greater mechanical pressure compared to hot pressing, quick cooling and heating, and pulsing current, that exposes the specimen to an electric field [207]. Diffusion and pores can be removed with the help of pressurized conditions [208]. The SPS method combines Joule heating, spark discharge, electrical diffusion, and plastic deformation to quickly and effectively sinter materials. Each active mechanism makes a unique contribution to various sintering phases [209]. The densification of nano-sized particles is not aided by heating rates attained exclusively by thermal approach, i.e., without use of applied field. The densification of the same particles is significantly influenced by electric fields [210,211]. The use of electric fields is not limited to uniaxial pressing when non-contact fields are present. The incorporation of a gas quenching mechanism into SPS was suggested to transform quick cooling SPS, which could subsequently be used for sinter hardening [212]. Powder metallurgy’s sinter hardening process requires quenching the finished products right away after sintering [213]. When the components are re-heated to the hardening temperature as opposed to traditional hardening [214], the components have superior dimensional stability and purity, as sinter quenching is carried out using gas rather than oil [215]. SPS is severely limited in its ability to increase sample sizes because of the power needed to reach higher temperatures, melting rates of dies, plungers [216]. As the component diameter gets closer to a few millimetres, it becomes unfeasible maintaining heating rate (>100 °C/min). It gets more difficult to maintain a consistent temperature throughout the sample with greater diameters [217].

### 3.3. Other Techniques Influencing Corrosion Resistance of Mg Alloys

Magnesium can be made resistant to corrosion via purification, chemical treatments, anodizing, lime treatment, protective coatings, surface modifications, adding new alloying components, and using cutting-edge metallurgical techniques [218]. In purification techniques, the elimination of impurities like Fe and Ni from Mg occurred which improves its corrosion resistance, as these contaminants (Fe and Ni) can induce galvanic corrosion. However, the complete elimination of impurities presents significant challenges [219]. The chemical treatment process entails the application of chemical agents to Mg surfaces to passivate them, thereby creating protective layers of phosphorous or chromate coatings [220]. However, there is a restricted resistance to corrosion, particularly in environments that are highly acidic or alkaline. Additionally, the chemicals employed, such as chromates, may pose toxicity risks and have detrimental effects on the environment [220]. Anodizing is a process that creates a thin layer of oxide on the surface of the metal by means of an electrochemical reaction. The thin oxide layer provides only a moderate level of protection. Susceptible to cracking and delamination when subjected to stress, MAO (micro-arc oxidation) enhances anodizing by creating thick, more resilient ceramic-like coatings that offer improved bioactivity and resistance to corrosion [221]. However, the lime (calcium hydroxide) is utilized in the treatment of Mg alloys, initiating a reaction that results in the formation of protective layers, thereby diminishing degradation. The lime treatments may create brittle surface layers that restrict the mechanical strength of the material, providing only transient protection [222].

Surface modifications, which include laser texturing and plasma spraying, among other processes, mainly involves the alternation of surface topography in order to improve corrosion resistance. But the implementation of specialized and costly equipment limits its usage. Further, the effectiveness of the surface modification approach is directly corelated with the obtained microstructure of material [223]. However, the introduction of alloying elements, such as zinc, aluminium, or rare earth metals, serves to improve corrosion resistance. The process of alloying can modify mechanical properties, resulting in diminished ductility or toughness. However, certain alloying constituents, i.e., aluminium and rare earth elements, raise issues regarding cytotoxicity in biomedical applications [224]. Moreover, the advanced metallurgical methods, such as rapid solidification and magnetron sputtering, are non-equilibrium techniques that enhance corrosion resistance through the refinement of grain structures or the formation of metastable phases [225]. But one of the most promising methods is micro-arc oxidation (MAO), also known as plasma electrolytic oxidation (PEO), which builds upon traditional anodizing techniques [78].

**Figure 6:**
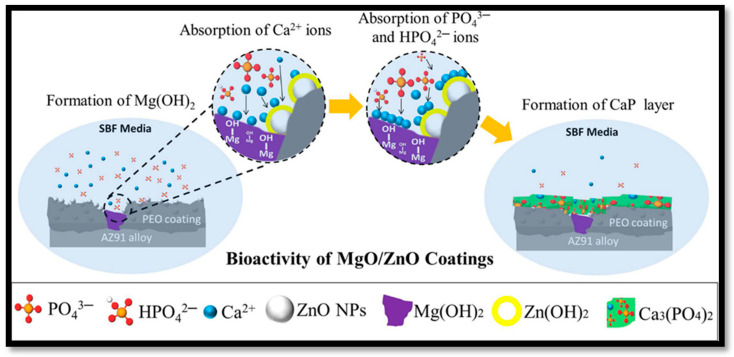
Ca_3_(PO_4_)_2_ layer development on the PEO coating after immersion in an SBF solution with and without ZnO nanoparticles [228].

MAO produces thick, ceramic-like oxide layers on Mg–Ti alloys, significantly improving corrosion resistance, biocompatibility, bioactivity, and antibacterial properties [226]. MAO coatings are particularly useful for biomedical applications due to their ability to incorporate bioactive elements, such as calcium and phosphorus, which enhance bone growth and osseointegration. The porous structure of MAO coatings facilitates the interaction between the implant and surrounding tissues, further improving implant integration [227]. Additionally, the functional coatings developed offer good antibacterial resistance, reducing the risk of infection around implanted materials. Khiabani et al. [228] employed ZnO nanoparticles to perform the PEO process on AZ91 Mg alloy for biomedical applications, including corrosion resistance and in vitro biodegradation. The alloy was treated with Ca_3_(PO_4_)_2_. The coating was subjected to bioactivity testing in a simulated bodily fluid (SBF) solution over two weeks. SBF increased the intensity of ZnO nanoparticles in the coating, resulting in increased calcium phosphate layer formation. Figure 6 depicts PEO coatings both without and with ZnO nanoparticles. Recent studies have demonstrated that MAO-treated Mg–Ti alloys exhibit superior performance in SBF as shown in Table 1. MAO coatings with enhanced bioactivity have been developed for orthopaedic and cardiovascular implants to reduce degradation and extend implant lifespan in vivo [229].

Non-equilibrium techniques, including mechanical alloying, magnetron sputtering, and rapid solidification, increased resistance to corrosion of Mg based materials [236]. Such synthesis procedures provide enhanced homogeneity, greater solid solubility of elements that alloy, the creation of new stages resistive to corrosion, and amorphization. The surface treatments of organic coatings, anodization, inhibitors, and electro- or electro-less plating are a few ways to stop corrosion [237]. Mg mechanical and physical properties are influenced by alloying elements through structural modifications [238]. Amongst several corrosion prevention approaches, alloying proved to be the most efficient affecting the entire material rather than just the surface, which is ineffectual once gone, such as through corrosion or scratches during implantation. Therefore, it became a pre-requisite to add suitable alloying element resisting corrosion with a sufficient amount of biocompatibility for Mg-based implantation.

### 3.4. Alloying Components in Mg-Based Materials

Focus is rapidly transiting to a recently prepared alloy designated with implantation in mind. It is typical practice to produce binary, ternary, and quaternary alloys. The elements used for alloying should produce stable oxide coatings that stop further corrosion. Among them is aluminium, which is used to enhance the mechanical properties and resistance to corrosion in a variety of commercial magnesium alloys [236,237,238]. Al-containing and non-containing magnesium alloys are the two categories into which they fall. To achieve a high degree of corrosion resistance, Mg-based alloys are single-phased, and chemically homogenized with passivating components in an appropriate amount [238]. Despite being one of the most researched magnesium alloys, AZ91’s main alloying component, aluminium, is known to be neurotoxic [239]. Prior to protective Al_2_O_3_ layer, AZ31 has good corrosion resistance and moderate strength which is not comparable to AZ91 [240]. Silver shows effectiveness in microbial activity and anti-inflammatory properties, Peng et al. included it in their Mg-Zn alloy [241]. The current pharmaceutical coatings on implants are to be replaced by this, as they are only capable of offering transient protection. Silver’s mechanical properties are enhanced by grain refinement.

Due to the proven biocompatibility of the alloying components, Rosalbino et al. examined ternary Mg alloy with Zn, and Mn implemented for biomedical purpose [242]. The increased Zn and Mn content in Ringer’s biological solution improved corrosion behaviour through stabilizing Mg(OH)_2_ layer on the surface through alloying metals. Mg-1.5Zn-1Mn alloys show high resistance to corrosion, low double-layer capacitance, and high charge transfer resistance, suitable for biodegradable implantation despite the lack of a biocompatibility study [243]. Spin-coating magnesium with PLGA in a simulated bodily fluid enhances corrosion potential, reducing corrosion current [244]. Further, when nano-hydroxyapatite particles are added to the coating more reduction is obtained. Surface treatment of AZ31B with extreme plasticity burnishing significantly increased corrosion resistance in NaCl solution [245]. A considerable reduction in grain and establishment of strong basal-texture grain orientation were linked to the improved corrosion behaviour. Residual tensions created during manufacturing may also have a beneficial influence on corrosion resistance [246]. The composites enhance the likelihood of localised corrosion while improving the resilience of magnesium against uniform corrosion. Yun et al. recommend utilizing reference electrodes to provide an external current as a cathodic protection technique for Mg implants [247].

Alloying elements mainly Al, Mn, and Zr improve mechanical and corrosion properties [248]. Mn binds iron and heavy metals, increasing yield strength and resistance to corrosion [249]. In the layered brucite structure, Mn oxide substitutes Mg ions blocking chloride ions, improving film protection, and limiting charge transfer. The high amounts of Mn weaken Mg’s corrosion resistance, cause cytotoxicity and even neurotoxicity; as a result, no more than 1 wt.% of Mn should be utilized [250]. Through grain refining, Zn strengthens Mg and reduces the corrosive effects of Ni, and Fe [251]. Although it is required for physiological purpose, an excessive level is harmful to cells. Excessive Zn addition, on the other hand, has a negative impact on corrosion behaviour [252]. Overdoses can be dangerous, hence the amount in Mg alloys should be kept to a maximum of 5% by weight to maintain them biocompatible and increase corrosion resistance. Zn and Mn, being the principal alloying elements, have considerable cytotoxicity, resulting in cell viability and genotoxicity [253]. Although Zr has been linked to cancers of the liver, lung, breast, and nasopharynx, which is biocompatible at low content. Mg alloyed with Zr promotes refining of grains [254]. Some rare earth elements (REEs) have been shown to be dangerous and should not be used in implant materials, rare earth elements (REEs) can assist magnesium resist corrosion. One characteristic of REEs (Nd, Er, La, and Ce) is a high degree of cytotoxicity, but thorium is quite toxic. REEs have an impact on inflammatory marker expression and apoptosis in vitro [255]. It is unknown if there are any adverse consequences in vivo. Ce has been shown to build up in the lungs, heart, liver, and bones. It can also have harmful effects on the body [256]. Er poisoning symptoms include lethargy, ataxia, and excessive sweating [257]. While yttrium may be dangerous, there is considerable disagreement over how Gd and Y affect corrosion [258]. Table 2 shows the general overview of few commonly bio-implantable materials that are used in combination with magnesium, along with their clinical tolerance and biocompatibility considerations.

Calcium is vital for healthy bone development. A disturbance in the body’s calcium balance can lead to severe health consequences [259]. The human body has a lot of calcium, especially in the bones. Mg–Ca alloys (low density of 1.55 g/cc) pertain to the density matching to natural bone, offering high strength, corrosion resistance, castability, and creep resistance. When Mg–Ca alloys were employed to replace osteochondral lesions in subchondral bone of distal femoral condyle of rat models, Han et al. discovered that they degraded quickly in vivo [260]. Following surgery, there were no infections or issues with wound healing. The presence of high quantities of bone formation markers was discovered. The yield strength and compressive strength was enhanced to 165 MPa and 254 MPa, respectively, by increasing the Ca content from 5% to 10% by weight. Hydrogen evolution was severe due to its rapid breakdown which is difficult to handle. Therefore, it is recommended to have less than 1 wt.% of Ca in Mg alloys for biomedical implantation purpose.

Sr is a bio-functional element enhancing bone repairing through osteoblast development [261]. Patients with postmenopausal osteoporosis may benefit from this increased bone cell development, which is osteoinductive and possesses great biocompatibility. At the same time, mechanical qualities improved with increased corrosion resistance with less than 2 wt.% of Sr. Sr influence on muscles and the heart has also been investigated for biomedical uses (Mg-Zr-Sr6, and Mg-Sr6). Corrosion resistance, mechanical characteristics, and biocompatibility are all improved in Mg alloys with the optimum weight fraction of Sr, and Zr [262]. Even dangerous components have been effectively employed with Mg alloys for biological applications, according to the researchers. But the future perspective focused on lowering corrosion rates and improving biocompatibility for Mg-based biodegradable implant materials.

According to Bowen et al., zinc is a better basis metal for bioabsorbable cardiac stents than magnesium because it integrates the benefits of iron, such as lifespan in vivo, with the safe breakdown of magnesium [263]. Zinc plays a role in nucleic acid metabolism, signal transduction, apoptotic regulation, and gene function and is required for fundamental biological function. In its ionic state, zinc has powerful antiatherogenic effects and functions as an antioxidant and endothelial membrane stabiliser [264]. A zinc stent’s systemic hazardous potential is negligible. As, Zn ions are transferred through living tissue, there should be no zinc enrichment that might cause cytotoxicity or necrosis during implantation, however cytotoxicity tests must be conducted. Zn–Mg alloys were studied for orthopaedic purposes but there were concerns about the toxicity of Zn implants [265]. Zinc has near-ideal biocorrosion properties. For up to six months following implantation, implanted wire samples were safely ringed by healthy arterial tissue, and the volume of linked tissue increased with time in vivo. Only a thin coating of zinc oxide formed in early stages of implantation; however, when localization caused rapid corrosion to progress, a phase rich in Ca and P appeared alongside the oxide, with zinc carbonate strewn on top [266]. The compact oxide layer’s development is impacted by local alkalization. With an elongation to failure of 60–80%, pure zinc exhibits high ductility; yet its tensile strength of just 120 MPa is inadequate for stent applications [267]. Iron and iron-based alloys have strong strength and modest corrosion rates, allowing them to withstand severe loads in bone repair and replacement [268]. Iron foams have been suggested to minimise the material’s high rigidity and avoid stress shielding. After allowing iron to decay in the tails of mice for several month, Mueller et al. discovered only minor inflammatory effects [269]. There were no cellular reactions to the extra iron, leading the researchers to believe that the iron corrosion breakdown products were metabolically inert.

### 3.5. Alternative Solution: PLGA (Polylactic Co-Glycolic Acid)

PLGA (polylactic co-glycolic acid) degrades without the use of enzymes by hydrolysis of ester backbone [270]. Polymer components, lactic acid and glycolic acid, are separated when degraded. As a result of this reaction, water and CO_2_ are produced. PLGA degradation by-products are easily absorbed into metabolic pathways and eventually eliminated, making PLGA biocompatible [271]. PLGA promotes osteoblast attachment, used in vitro and in vivo conditions for creating scaffolds. PLGA acts as a corrosion-resistant covering for magnesium [272]. Research conducted in vitro revealed that PLGA coating over iron enhanced the disintegration metal corrosion rate. To alter PLGA’s degradation properties, the hydrophilic for quicker breakdown and hydrophobic for slower deterioration was preferred [273]. Degradation is also slowed by higher molecular weight, Yoo et al. found PLGA broke down more slowly in an acidic atmosphere than it did in a PBS solution with a higher pH level [274]. The pH shift resulted in decreased mineral concentration, collagen synthesis, and glycolysis. Regarding biodegradable implant materials, the impact of pH shift on cell activity is important as polymers that degrade are known to produce an acidic milieu during breakdown. The results also demonstrate that biologic activity and cell behaviour can be impacted by the area’s degrading polymers.

The acidic by-products of polymer degradation are thought to be the cause of the inflammatory reaction [275]. The pH level affects the growth of osteoblasts. The composition of extracellular fluids has an impact on bone mineralization and thus repair. The pH level affects the precipitation of Ca–P salts [276]. The tissue pH level affects mineralization and bone healing. In vivo, even a modest decrease in extracellular pH has an effect on osteoblast function. Acidic PLGA degradation products may alter the pH, inhibiting bone metabolism and slowing healing. The acidic breakdown products of PLGA disintegration have been shown to cause considerable tissue damage and inflammation in animal tests [277]. Kohn et al. [278] found that the bone marrow cell stromal cells are significantly affected by reducing extracellular pH. Alkaline phosphatase activity, collagen synthesis, and collagen gene expression were all altered by a low pH. One possible way to mitigate the detrimental effects of PLGA breakdown is to balance the pH drop by adding basic salts or applying a layer of carbonated apatite minerals to the surface [279]. The addition of basic salts, such as calcium carbonate, significantly increased the biocompatibility of biodegradable polymers.

Mg–Ti particles were employed as reinforcing agent in PLGA to bulk application to serve as an alternative [280]. When PLGA breaks down in the body, the pH dropped, damages surrounding cells that are exposed to the breakdown of the products’ acidic nature. Magnesium/Mg–Ti alloys are added to PLGA controlling pH and improving the material’s biocompatibility [281]. Ultimately, PLGA is not appropriate for load-bearing applications due of its low strength. To improve the mechanical properties and increase the biological applications of PLGA-metal particle composites, stronger metal particles might be included. The modulus of PLGA is low, which could be enhanced by introducing a material with a greater modulus. The use of hydroxyapatite and polymers as PLGA reinforcing materials has been studied. Wu et al. [282] experimentally verified that the reinforcement of PLGA to AZ31 metal fibres enhances ultimate tensile strength, and elongation of PLGA. Mg serves as a potential option for disposable metallic implantation because of its mechanical characteristics, which are more similar to those of bone than those of steel or titanium alloys [283]. The addition of magnesium accelerated the decay of PLGA, and those larger concentrations of the magnesium alloy led to higher rates of corrosion.

## 4. Titanium and Corrosion Characteristics

On the surfaces of dental implants and abutments, many types of corrosion have been seen. When the surface of metal is exposed to cathodic reactants, uniform corrosion occurs leading to corrosion nucleus [284]. Pitting corrosion is localised corrosion with voids on the surface, known as pits, more harmful than general corrosion due to the difficulty in detecting, predicting, and designing pits [285]. Pitting corrosion occurs on titanium dental implants when the titanium oxide thin coating breaks down locally in the oral environment [286]. Galvanic corrosion happens when two different metals in an electrolytic solution come into close contact [287]. On the inner contacting surfaces of titanium implant-abutment contacts, galvanic corrosion occurs. When other basic metal alloys are utilised for abutments, the surface damage can be enhanced (e.g., stainless-steel and CoCr alloys). Crevice corrosion is a type of localised surface deterioration that occurs at the contacting surfaces of implants, abutments, and prosthetic devices, resulting in restricted oral fluid entrance and egress as well as oxygen depletion [288]. Due to the presence of free H+ ions in the medium, physiological fluid becomes acidic in these confined contacting zones, lowering pH levels [289]. Furthermore, the dynamic mastication loading that causes micro-motion at the implant-abutment connections enhances contacting surface wear and premature breakdown of the titanium oxide thin layer [290]. As a result of the synergistic action of corrosion and micro-motions, fretting corrosion results in structural changes and wear on the surfaces. This action is the major cause of metal release into the surrounding tissues.

The titanium oxide thin film shields the titanium implant surface from the corrosive oral environment’s reactive ions [291]. Due to the microstructure of titanium and Ti alloy, the spontaneously passive film contains certain inclusions and discontinuity patches, despite its strong corrosion resistance. As a result, the quality of the native oxide film is influenced, and this could be the first point of corrosion. Corrosive chemicals such as fluorides, lactic acid, carbamide peroxide (urea peroxide), and hydrogen peroxide erode the protective titanium oxide coating [292]. When the HF concentration is greater than around 30 ppm, the titanium passivation film is destroyed, according to Nakagawa et al. [293]. As a result, corrosion in fluoride solutions is influenced by the pH and the generation of HF caused by the dissociation of NaF at high concentrations, or by the bonding of H^+^ and F in low pH solutions. A high F concentration paired with a low pH level can increase the chemical reactivity of titanium [294]. Localized titanium corrosion was identified in fluoride solutions containing 227 ppm F at a pH of 4; however, the F level at a pH of 5.5 was insufficient to disturb the titanium passive coating. In the presence of a high concentration of H^+^ from the acidic medium, the TiO_2_ layer is changed, resulting in the creation of hydrated Ti oxides as Ti(OH)_3_ and the subsequent release of Ti ions and TiO_2_ ultra-fine particles into the environment [295]. In inorganic buffer solutions, Cp Ti tends to repassivate faster than Ti6Al4V. When compared to the alloy, electrochemical experiments revealed a lower critical current density and better catalytic activity for the hydrogen evolution reaction on cp-Ti [296].

Titanium corrosion is usually not accelerated when it is combined with different metals [297]. The only exception is in strongly reducing conditions, when titanium corrodes rapidly and is difficult to passivate. In its natural state, titanium is impacted positively by materials with higher noble (positive) corrosion potentials. When combined with titanium, graphite, and various precious metals (such as platinum, palladium, ruthenium, iridium, and gold), it provides anodic protection by maintaining the titanium oxide coating at higher noble potentials [296,297]. Titanium has a noble corrosion potential under normal passive conditions, but it is similar to stainless steel or nickel-based alloys in this regard. As long as inert conditions hold for the alloys involved, the tiny variances between these passive engineering alloys often entail low galvanic interactions and good galvanic compatibility. Titanium and its alloys are among the metals that are most corrosion-resistant, with galvanic, pitting, crevice, and fretting corrosion almost absent [298]. Titanium, like aluminium, creates a corrosion-resistant coating that recovers after being damaged. However, titanium’s corrosion layer is more efficient than aluminium, and its mechanical qualities are superior to aluminium’s [297,298,299]. Table 3 summarizes the experiments on titanium corrosion in a simulated environment. Furthermore, there is fear that aluminium ions may cause Alzheimer’s disease which restrict the usage of element during implantation. Because magnesium and titanium are both biocompatible on their own, integrating them into an alloy could result in a new biocompatible material. Titanium can enhance the inactivity of magnesium by acting as a strong passivating element [299].

### Cytotoxicity and Biomedical Implantation

The first stage in determining a possible new implant’s biocompatibility is to evaluate its cytotoxicity, preferably on wound site cells linked to implant material [302]. Metal ions emit elicit biological responses in surrounding tissues over short or long periods of time. As a result, a metal’s wear resistance and corrosion rate affect its toxicity in addition to its composition and the toxicity of its constituent elements. Cytotoxicity can manifest itself in a variety of ways, including morphologically visible cell destruction, cytosolic chemical release, altered proliferation, and changed metabolism [303]. To quantify cell attachment in implantation, in vitro testing is required. The cells will either migrate away from the implant material or die from necrosis if they are unable to attach to it [304]. In necrosis, the cell loses integrity and dies instantly due to cell lysis; whereas, in apoptosis, the cell stops developing and dies gradually. Metals corrode when they come into touch with biological systems [305]. High ion concentrations have long been linked to corroding implants, according to research conducted on animals. Ion-protein complexes formed by the liberated metal ions and particles have a strong immunogenic potential [306]. Degradation can wreak havoc on the implant’s mechanical qualities, weakening it and allowing more ions or particles to leak out. The ions themselves, debris, organometallic complexes, an organic metallic salt, and oxides all have the potential to cause allergic reactions [307]. Metal ion or particle phagocytosis can cause cell-mediated allergic responses. As a result, corrosion products might be immunogenic. Toxicity, implant loosening, chronic inflammation, aseptic osteolysis, hypersensitivity responses, and carcinogenesis are risks with implant material [308]. It has been demonstrated that even non-cytotoxic content (chromium, titanium, cobalt) may stimulate osteoclasts, which in turn can lead to bone resorption [309]. Even non-allergenic substances can disrupt bone metabolism by causing local irritation in this way. It has been demonstrated that the magnesium ions that are produced when magnesium corrodes are not only safe for surrounding tissue cells, but are beneficial to them. Magnesium is biocompatible and does not cause harmful systemic responses; nevertheless, its effects on the surrounding environment during implantation needs further exploration [310]. Unlike systemic biocompatibility, Mg ions surrounding wound and pH variations revealed substantial effect on implantation.

Gao et al. [311] performed cytotoxicity experiments on extracts from corroded samples and Mg in direct contact. Extracts were produced from samples that had been corroded in a medium for 24 h at 37 °C. For the 6-day cytocompatibility assays, the pre-osteoblastic cell line MC3T3-E1 was employed. Osteoblast cell development was reported to be quicker than that of control cells cultured in only media. In comparison to the control, viability was reduced by 28–73% at Mg ion (505–374 mg/L). Pichler et al. [312] examined survival and metabolism of plate chondrocytes and MG63 osteoblasts in Mg alloy (WZ21 and ZX50). In terms of cell viability, cell proliferation, and metabolic activity, a slowly deteriorating WZ21 outperformed the other alloys studied. In MG63 cells, ZX50 eluate caused an increase of osteogenic markers. This is a good thing because high levels of alkaline phosphatase suggest bone growth [313]. Articular chondrocytes were found to have a positive effect on proliferating and re-differentiating [314]. Magnesium enhances cell proliferation, matrix formation, and chondrogenic marker expression in these cells in proportion to ion concentration [315]. Based on the research outcomes, the alloys of magnesium absorbed by osteoblasts promote plate chondrocytes which is effective for biodegradable implantation.

When human osteosarcoma MG63 cells and human oral epithelial KB cells are cultured on magnesium materials, Wang et al. discovered that they are inhibited [315]. This suggests that establishing an alkaline environment in the vicinity of cancer cells could be an approach to treat bone cancer. Bone cancer cells, known as osteosarcoma U2-OS cells, are cytotoxically affected by pure magnesium extracts [316]. Since the effect was not observed with a rise in Mg ion concentration, it may have resulted from a rise in pH levels brought on by magnesium corrosion. Several studies have shown that magnesium salts and several magnesium alloys have favourable biocompatibility with bone cells [316,317,318,319]. Magnesium improves bone cell adherence to biomaterials, and successfully implemented in osteointegration [319]. Around the magnesium implant, the bone mass had also risen. Magnesium is associated with both effects because it activates bone cells. Di Virgilio et al. studied the cytotoxic impact of Mg on mouse osteosarcoma UMR106 cells [89].

In dentistry, orthopaedics, and trauma applications, Ti-based materials are often utilized in biomaterials because of their rigidity, low intrinsic toxicity, low reactivity with biomolecules in comparison to other biomaterials, and have exceptional production capabilities [320,321] Titanium particles did not cause any issues, even if they managed to escape the implant site. Titanium particles were thought to have been caused by mechanical wear during implant insertion rather than corrosion when they were found distant from the implant site [322]. Commercially pure titanium does not cause anaphylaxis or hypersensitivity. All current imaging methods are compatible with titanium and titanium alloys, which are non-toxic. As titanium has a track record of success in dental implants, it is really among the most biocompatible metals. Titanium implants have the unique property of fusing to hard and soft tissue surface rather than creating fibrous capsules around the implantation [323]. Conversely, nanoscale titanium wear particles contribute to the start and development of aseptic release by inducing inflammatory reactions that ultimately lead to osteolytic activity. Despite the widespread belief that titanium implants are biocompatible, there is a possibility that the ions they release might cause allergic responses or negatively impact the surrounding tissue. In bulk titanium alloys including ions, Li et al. looked at the harmful effects of titanium and other widely used alloying elements [324]. The researchers discovered that the SaOS-2 cell line’s osteoblast-like human osteosarcoma cells propagated effectively on bulk titanium, indicating great biocompatibility. The viability of the cells was considerably reduced when cultivated from extracts generated by incubating titanium powder in a medium.

Ti ions influenced osteoclast precursor by inducing the production of certain cytokines. Precursors of human osteoclasts can develop on titanium surfaces and mature into osteoclasts [325]. The mature cells corrode the metal directly and release titanium ions. These ions are taken up by the osteoclasts, but they are then released. These ions are thought to promote inflammation and stimulate osteoclastic differentiation. They’ve also been related to osteolytic lesions in periprosthetic bone, leads loosening of implantation. A Ca^2+^ receptor located in the plasma membrane of osteoclasts is used as a feedback system for bone resorption [326]. Mg^2+^ exposure can decrease bone resorption, since the receptor can bind a variety of divalent and trivalent cations [326]. Thus, slow down the bone resorption rate which will ultimately stimulate bone growth during the initial healing period.

The re-modelling of immature bone into mature was impeded, contingent upon the ion concentrations released by the implant material [327]. In osteogenic cells generated from bone marrow, Puleo and Huh evaluated the acute toxicity of different metal ions widely utilised in orthopaedic implantation [328]. Only the highest quantities (25 and 50 ppm) of Ti^4+^ ions were hazardous after 48 h of exposure. Time-dependent cytotoxicity resulted in irreversible toxic effects within 3–6 h after ion exposure. Additionally, titanium ion at sublethal levels have been demonstrated by Thompson and Puleo to affect osteoblastic cell growth and function in vitro. Ti^4+^ ions inhibited the production of a mineralized matrix and the release of osteocalcin by cells while having no effect on alkaline phosphatase secretion or cell proliferation [329]. This may indicate that osteoblast differentiation is being disrupted by titanium ions. Sayes conducted a study on the cytotoxic consequences of nanoscale titanium on human lung epithelial cells and human dermal fibroblasts [330]. Only very high doses of 100 g/mL caused cytotoxicity and inflammation. The effects of exposure duration grow with the strength of the cellular response. Material created a reactive species under UV irradiation tied to the harmful effect. Fischer et al. [331] found that the inclusion of Mg ions to cell culture interferes in stained mechanism, producing inaccurate findings, meaning that MTT, a well-known cytotoxicity staining method, cannot be used to analyse magnesium alloys. Titanium seems to erode alongside or separate from the substance when the surrounding magnesium dissolves, but much more slowly than magnesium. On titanium, human osteoclast precursor cells can proliferate and develop into mature cells, as stated by Cadosch et al. [270]. Even titanium as a metal, which is considered to be bioinert, could be eliminated from the location of implantation during this process. Therefore, Mg–Ti is a biodegradable material utilized in a biodegradable implant, imparting sufficient biocompatibility. An electrochemical scan and a hydrogen ions evolution test showed alloy with the greatest magnesium ion concentration had significantly enhanced corrosion resistance.

## 5. Conclusions and Future Outlook

Metals, ceramics, polymers, and composites are the four major forms of biomaterials used for implantation. Stainless steels, titanium, and cobalt-chromium-based alloys are commonly used metallic biomaterials. Metallic materials other than ceramics or polymers are crucial in providing a good healing rate to tissue, replacing diseased or damaged bone tissue, suitable for load-bearing applications accommodating high mechanical strength and fracture toughness. Hence, metallic implant materials are employed in a wide range of applications in biomedical applications. The implant material must possess sufficient biocompatibility, bio-activeness, and biodegradability. Biodegradable implants provide the remodelling of bone, tissue healing rate, tabulating the injured bone, and restricting the chances of a second surgery, relying on polymers and metals for biodegradable implant materials that are mechanically strong and have good corrosion resistance. Magnesium is considered bioactive, biodegradable, biocompatible, and resembles the elastic modulus of natural bone. The low elastic modulus of Mg-based materials helps in reducing the stress shielding effect, which is a major problem with stainless steel, cobalt-chromium alloy, and titanium-based implant material. Due to the high value of the elastic modulus, most of the stress is acting on the bone, hindering the new bone stimulation and remodelling. But corrosion of Mg and its alloys are the major areas of concern when dealing with biomedical implantation. Enhancing the corrosion resistance of Mg-based alloys in SBF (simulated body fluid) without affecting its biocompatibility is a serious concern. Titanium is regarded as a suitable reinforcing agent that improves corrosion resistance of Mg based material, maintaining biocompatibility.

Biomaterials promote the interaction of cells enabling the regeneration of new bones. Mechanical stresses promote cell function and impact the adhesion of integrins on cell surfaces with respect to the substrate. Mechano-transduction is the process by which cells turn stresses into biological signals, initiated by structural stresses or fluid shear stresses generated in the materials. Pore size, distribution, shape, hydrophobicity/hydrophilicity, time-dependent deformation, chemical functionality, pH change, temperature and stress, micro- and nanoscale surface topography, and mechanisms of biodegradation are the factors affecting implantation. Artificial implant biomaterials are implemented to relieve pain and restore dysfunctional tissue performance. Stainless steel involves pins, wires, screws, plates, intramedullary nails or rods, among other items, employed in internal fixation devices due to its high strength. Biodegradable implantation is preferred for a rapid tissue healing rate to bone tissue along with the regeneration of tissue bone. Magnesium (biodegradable material) is a promising material for biomedical applications offering high cytocompatibility and superior mechanical properties. Mg is employed in bone and cardiovascular applications for restoring fractures. Magnesium-based implants provide initial stability and load-bearing support to the implant before it is degraded in vivo, thereby terminating the need for additional surgery after implantation.

Magnesium (Mg) is susceptible to corrosion, which limits its use in biomedical implants and structural applications. Techniques to enhance Mg’s resistance include purification, chemical treatments, anodizing, lime treatment, protective coatings, surface modifications, alloying, and advanced metallurgical processes. Purification eliminates impurities like Fe and Ni, improving corrosion resistance. Chemical treatments passivate Mg surfaces, creating protective layers of phosphorous or chromate. However, these methods have limitations, especially in acidic or alkaline environments. Anodizing creates a thin oxide layer, but MAO (micro-arc oxidation) enhances it by creating thick, more resilient ceramic-like coatings. Surface modifications, like laser texturing and plasma spraying, alter surface topography, but their effectiveness depends on the material’s microstructure. Alloying elements like zinc, aluminium, or rare earth metals improve corrosion resistance, but raise cytotoxicity concerns. Advanced metallurgical methods, like rapid solidification and magnetron sputtering, refine grain structures or form metastable phases. MAO coatings are particularly useful for biomedical applications, incorporating bioactive elements like calcium and phosphorus, enhancing bone growth and osseointegration.

Mechanical alloying is used to create alloys and advanced materials at room temperature. Initially, the term milling applied to dissolving large particles into smaller ones, but now creates new phases and materials with enhanced physical and mechanical properties. However, the non-equilibrium approach of high-energy ball milling for nanoscale micro-structured materials has received considerable attention. Further, SPS works better than hot pressing and hot isostatic pressing for sintering. SPS has the advantages of regionally shattering the oxide layer, permitting adhesion between particles through the oxide layer’s holes, and enabling full density sintering of even difficult-to-treat materials using traditional sintering techniques. Several factors make SPS a particularly quick densification method, including quick heat transfer, a greater mechanical pressure compared to hot pressing, quick cooling and heating, and pulsing current, that exposes the specimen to an electric field. Diffusion and pores can be removed with the help of pressurized conditions. However, under physiologically simulated circumstances, Mg–Ti alloys exhibit superior corrosion resistance and cytotoxicity that is equivalent to Mg. The reduction in corrosion rate was accountable to titanium and magnesium alloy. The slower rate of corrosion is most likely the cause of the decreased cytotoxicity. More investigation on SPS consolidation is required to ascertain whether maximum density may be reached with a different combination of parameters. Due of the scaling limitations of SPS, it is beneficial to concurrently explore other non-equilibrium consolidation techniques. Understanding the interaction between corrosion and cyclic loading is important to understand if the material can withstand the stresses during fracture healing. Corrosion behaviour of Mg and Mg–Ti in SBF is the major barrier to the practical application of alloys made of magnesium, which otherwise offer exceptional qualities to be utilized as temporary implantation in physiological situations. The benefits of titanium alloying were assessed using pure magnesium as a control.

## Figures and Tables

**Table 1:** Recent studies in Micro-arc oxidation (MAO) employed in functional coatings with higher corrosion resistance, biocompatibility, bioactivity, and antibacterial ability.

S. No.	Researcher	Results/Outcomes	References
1.	Zhu et al.	Calcium ions enter MAO coatings through diffusion and electromigration, with proper Na12Phy improving calcium amount. Surface morphology influences corrosion resistance, while Ca(H_2_PO_4_)_2_·H_2_O enhances calcium amount.	[230]
2.	Shi et al.	Near-neutral solutions were used to prepare Ca-P MAO coatings on Mg alloy, with IP6 enhancing calcium content and corrosion resistance, while EDTA-CaNa_2_ corrosively affects magnesium alloys.	[231]
3.	Qiao et al.	The orthogonal experiment investigated the entrance mechanism of calcium into anodic coatings through diffusion and electromigration, achieving the influencing sequences of factors on Ca and P contents.	[232]
4.	Li et al.	Multipurpose MAO coatings on Mg alloys showing anti-bacterial properties	[233]
5.	Liu et al.	Optimizing magnesium implant corrosion resistance using doped MAO coatings	[234]
6.	Wang et al.	Improved MAO coatings with regulated porosity for use in biomedicine	[235]

**Table 2:** Clinical Tolerance of Bio-implantable Materials Used with Magnesium (Mg).

Material	Clinical Tolerance	Key Properties	Limitations
Titanium	High biocompatibility, excellent corrosion resistance	Promotes osseointegration	Higher elastic modulus than bone (stress shielding)
Zinc	Moderate biocompatibility, biodegradable	Anti-inflammatory, antioxidant properties	Cytotoxic in high concentrations
Calcium	Essential for bone growth; well-tolerated in low amounts	Promotes osteogenesis	High concentrations may cause rapid degradation and hydrogen evolution
Aluminium	Good corrosion resistance in some alloys (AZ91)	Improves mechanical properties	Potential neurotoxicity
Silver	Anti-bacterial properties, moderate biocompatibility	Reduces infections	Cytotoxic in high concentrations; short-term protection only
Manganese	Moderately biocompatible, enhances mechanical strength	Improves corrosion resistance	Excess Mn content can cause cytotoxicity and neurotoxicity
Rare Earth Elements (REEs)	Varying biocompatibility, generally improves corrosion resistance	Promotes long-term stability	Cytotoxicity concerns (Gd, Y), may accumulate in tissues
Polylactic-co-glycolic acid	Excellent biocompatibility, biodegradable polymer	Controls degradation rate of Mg implants	Requires precise processing for uniform coatings

**Table 3:** Titanium/Magnesium corrosion in a simulated environment are summarized.

Researcher	Material	Results	References
Royhman et al.	Ti-6Al-4V disk	Nicotine appeared to reduce local rusting at certain concentrations. However, it slowed the growth of passive films.	[300]
Bhola et al.	Ti6Al4V, Ti15Mo	On Ti6Al4V alloy, listerine shows an increase in corrosion rate, while on Ti15Mo alloy, it shows a decrease in corrosion rate.	[288]
Sridhar et al.	Large grit, acid-etched cp Ti implants	Discoloration, fracture, surface delamination, and fatigue cracks were seen on the surface, indicating surface degradation. Micro-pits are a type of surface degradation.	[301]
Nakagawa et al.	Ti-based implant material	HF concentration exceeds 30 ppm, titanium passivation film is destroyed	[293]

## Data Availability

Not Applicable.
